# A Study of A1 and A2 Subtypes Among Whole-Blood Donors With Blood Groups A and AB at the Blood Center of a Tertiary Care Institute in Chhattisgarh

**DOI:** 10.7759/cureus.57013

**Published:** 2024-03-27

**Authors:** Minal Wasnik, Saurabh Lahare, Ramesh Chandrakar, Nitin Kumar Kashyap

**Affiliations:** 1 Transfusion Medicine and Blood Bank, All India Institute of Medical Sciences, Raipur, Raipur, IND; 2 Transfusion Medicine, All India Institute of Medical Sciences, Patna, Patna, IND; 3 Cardiothoracic Surgery, All India Institute of Medical Sciences, Raipur, Raipur, IND

**Keywords:** blood donor, aend, aint, subtype, a2, a1, blood group

## Abstract

Introduction: The ABO blood group shows various subtypes due to the heterogeneity of A and B alleles. The frequency of these subtypes varies in different populations. Studies related to the frequency of subtypes of blood groups A and AB are lacking in this region. So, we planned this study to estimate the prevalence of A_1_ and A_2_ subtypes among the healthy blood donor population.

Materials and methods: This was a prospective study performed in the blood center of a teaching hospital in the Chhattisgarh state. Healthy whole-blood donors were included in the study after written informed consent. The conventional test tube method was used for performing forward and reverse blood grouping. Testing with anti-A_1_ and anti-H lectin was performed in blood groups A and AB. Additional tests such as saliva testing for secretor status and adsorption-elution were performed if needed.

Results: Four thousand one hundred twelve donor samples were studied, out of which 1170 showed A antigen. Among 1170 samples, 74.6% were blood group A, and 25.4% were AB. Among blood group A, 92.3% were A_1_ and 3.3% A_2_,_ _and the rest were other subtypes, while in AB, it was 85.2% A_1_B and 14.8% A_2_B. Two cases of anti-A_1_ antibodies were also noted, which were clinically insignificant.

Conclusion: We observed a significantly higher proportion of A_2_B than A_2_ in our study population. We also found a large proportion of A_int_ in the study participants. Testing with anti-A_1_ and anti-H lectin is recommended in blood groups A and AB to determine various subtypes and prevent any incompatibility.

## Introduction

During the 1900s, Karl Landsteiner discovered the ABO blood group system, which has become the most important system for clinical transfusion medicine. Individuals with blood groups A, B, AB, and O have red blood cells (RBCs) that exhibit A, B, AB, or no antigen, as well as a serum that contains naturally occurring anti-B, anti-A, no antibodies, or both anti-B and anti-A. It is these naturally occurring antibodies that hamper the blood group-incompatible transfusion or transplantation. ABO antigens are produced by adding terminal sugar to an oligosaccharide H chain using blood group-specific transferases, which transfer N-acetyl-D-galactosamine or D-galactose sugar to form either A or B antigens, respectively [1].

Various ABO subtypes have been observed due to the heterogeneity of A and B alleles. These subtypes may present as discrepancies during immunohematological testing. Variable serologic reactivity with human polyclonal anti-A, anti-B, and anti-AB reagents is observed in these subtypes. A_1_ and A_2_ are the major subtypes encountered in blood group A, which differ both qualitatively and quantitatively from each other. A_1_ red cells have 8.1-11.7×10^5^ antigenic sites as compared to 2.4-2.9×10^5^ antigenic sites on A_2_ red cells. Both A_1_ and A_2_ show strong agglutination by anti-A antiserum. However, anti-A_1_ lectin of *Dolichos biflorus* agglutinates A_1_ red cells but not A_2_ red cells. As the A_2_ phenotype reflects the inefficient conversion of H to A antigen, they show increased reactivity with the anti-H lectin of *Ulex europaeus*. A_1_ is the most common subtype (80%), followed by A_2_ [2]. The lesser observed weak subtypes of blood group A include A_3_, A_end_, A_x_, A_m_, A_y_, and A_el_ observed in <1% [1]. The prevalence of A subtypes varies in different populations and different places. In some populations, such as blacks and Japanese, the frequency of the A_2_B phenotype is significantly higher than the expected frequency based on the frequency of the A_2_ phenotype [3,4]. In the southern part of India, the prevalences of A_1_, A_2_, and other weak subtypes were reported to be 98.14%, 1.85%, and 0.01%, respectively [5]. A hospital-based study performed in Northeastern India showed A_1_ to be 98.3% with the rest being A_2_ and weak subtypes [6].

On reviewing the literature extensively, no similar study was found regarding the frequency of subtypes in this region. So, we planned this study to determine the prevalence of A subtypes in donors with blood groups A and AB. A part of this study project was previously presented as an abstract in the TRANSMEDCON 2022 conference.

## Materials and methods

This prospective study was conducted in a blood center affiliated with the Department of Transfusion Medicine of a tertiary care teaching hospital in Chhattisgarh between July 2021 and December 2022. The study included 4112 donor samples. After receiving approval from the Institute Ethics Committee of the All India Institute of Medical Sciences, Raipur (approval number: AIIMSRPR/IEC/2O21/699), the study was initiated. Departmental standard operating procedures were followed in the selection of blood donors, which was based on the Drugs and Cosmetics Act, India, with the latest amendments [7]. The study included whole-blood donors who consented to participate. We excluded apheresis donors and therapeutic phlebotomies.

The blood group of the donors was tested by conventional tube technique (CTT). For this purpose, donor blood samples were collected in a vial of ethylenediaminetetraacetic acid (EDTA) at the time of blood donation, after getting informed written consent. The blood group was determined by forward and reverse grouping techniques as per departmental standard operating procedure. Monoclonal antisera anti-A, anti-B, anti-AB, and anti-D (Tulip Diagnostics, Verna, India) were used for forward grouping, and in-house fresh pooled A, B, and O cells were used for reverse grouping. A trained technician performed all the procedures under the supervision of a medical officer following the manufacturers' instructions. Blood groups were determined based on the agglutination pattern in forward and reverse grouping. When the forward and reverse grouping showed coherent results, then only the blood group results were considered valid. Any discrepancy in the forward and reverse grouping was resolved prior to validating the blood group results.

**Figure 1:**
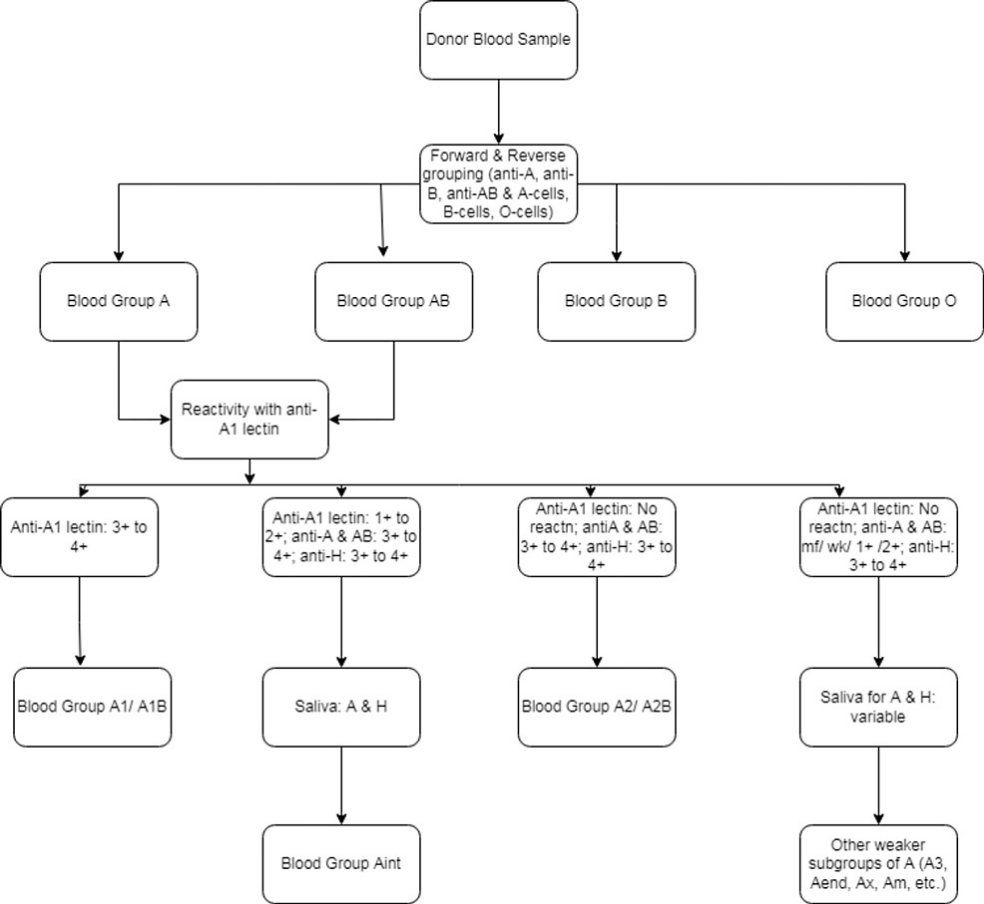
Workflow for the testing of subtypes of blood groups A and AB reactn, reaction; mf, mixed field; wk, weak

To classify the samples of blood groups A and AB according to their subtype (A_1_, A_2_, and other subtypes), anti-A_1_ lectin was used. Macroscopic agglutination with monoclonal anti-A and no agglutination with anti-A_1_ lectin were considered as A_2_ subtypes. We also tested O blood groups with anti-A and anti-B antisera to identify weak subtypes of A [8]. The plasma of all the subtypes other than A_1_ was further tested with A_1_ cells to detect anti-A_1_ antibodies. If detected, the thermal amplitude of anti-A_1_ antibodies was determined at 4°C, room temperature, and 37°C. Whenever needed, additional testing with anti-H lectin, saliva testing for secretor status, and adsorption-elution studies were performed according to departmental standard operating procedures based on procedures described elsewhere [9]. Figure 1 depicts the workflow for testing samples. In Microsoft Excel (Microsoft Corp., Redmond, WA) spreadsheets, donor demographics, and immunohematology testing data were entered and analyzed.

## Results

The study included 4112 accepted whole-blood donors. The majority (97.5%) of the donors were males (n=4088). Females accounted for only 2.5% as the majority were found ineligible due to low hemoglobin and low weight. Of the donors, 53.5% were 21-30 years, followed by 27.7% in the age group of 31-40 years. We also had 10 participants of >60 years. Of all the donor samples studied, blood groups A, B, AB, and O were found in 21.23% (n=873), 34.46% (n=1417), 7.22% (n=297), and 37.09% (n=1525), respectively. A antigen was present in 1170 donor samples (Table 1).

**Table 1:** Frequency of blood groups A and AB

A antigen in ABO blood group	Frequency (%)
A	873 (74.6%)
AB	297 (25.4%)
Total	1170 (100%)

On testing with anti-A_1_ lectin, A_1_ and A_1_B subtypes were found in 806 and 253 samples, respectively, while A_2_ and A_2_B were found in 29 and 44 samples, respectively (Table 2). We found 37 samples showing intermediate reaction (1-2+) with anti-A_1_, for which repeat testing was done with fresh blood samples using antisera of different lots at different temperatures and saliva testing for secretor status. Based on the immunohematological workup, these 37 samples were labeled as blood group A_int_. We also encountered one blood group A_end_ (Table 3). Anti-A_1_ antibody was found in one case in each of blood groups A_int_ and A_2_B, both of which were reactive at room temperature and 4°C but not at 37°C.

**Table 2:** Subtypes of blood groups A and AB

ABO blood group	Total number	Subtypes	n (%)	Anti-A_1_ antibody
A	873	A_1_	806 (92.3)	-
		A_2_	29 (3.3)	-
		A_int_	37 (4.2)	1
		A_end_	1 (0.2)	-
AB	297	A_1_B	253 (85.2)	-
		A_2_B	44 (14.8)	1

**Table 3:** Immunohematological workup for blood groups Aint and Aend wk, weak; mf, mixed field

Forward grouping						Reverse grouping			Auto-control	Saliva testing	Blood group
Anti-A	Anti-B	Anti-AB	Anti-D	Anti-A_1 _lectin	Anti-H lectin	A_1 _cells	B cells	O cells			
4+	0	4+	4+	1+	3+/4+	0	4+	0	0	A and H	A_int_
wk/mf	0	wk/mf	4+	0	4+	0	4+	0	0	H	A_end _(adsorption-elution showed the presence of A antigen)

We observed that the proportion of A_2_B (14.8%) among AB was higher than A_2_ (3.3%) among blood group A. On statistical analysis, this difference was found to be of statistical significance (p<0.05). The ratio of A_2_/A_1_ is 0.04, while A_2_B/A_1_B is 0.17.

## Discussion

The frequency of ABO blood groups varies among different populations. A phenotype is found mainly in Northern and Central Europe, while B phenotype is most frequent in Central Asia. Blood group O is the most frequent phenotype globally [10]. Our study included 4112 participants with 1170 having A antigen. Among these, 74.6% were A, and 25.4% were blood group AB, which is similar to the studies performed in North Karnataka [11], as well as Sudan [12].

Blood group A is mainly divided into A_1_ and A_2_ subtypes based on reaction with anti-A_1_ lectin. However, there are several other subtypes such as A_3_, A_int_, A_end_, and A_m_. A_2_ and A_2_B subtypes are usually less common. Our study findings of A_1_ being more common than the A_2_ subtype are similar to studies conducted in South Indian and the Sudanese population [5,12]. We found that the prevalence of A_1_ and A_2_ in blood group A was 92.3% and 3.3%, respectively, whereas that of A_1_B and A_2_B in blood group AB was 85.2% and 14.8%, respectively. In their study, Giriyan et al. observed the prevalence of A_1_ and A_2_ to be 98.90% and 1.10%, respectively, and that of A_1_B and A_2_B to be 89.70% and 10.30%, respectively [11]. Our study findings are similar to that of a pilot study performed by Kumar et al., which showed A_2_ and A_2_B to be 4.1% and 19.2%, respectively [13].

We observed that the frequency of A_2_B in blood group AB as compared to A_2_ in blood group A is higher, which was statistically significant. Our findings are similar to studies conducted on blacks and the Japanese population [3,4]. A study conducted by Shastry and Bhat in South India also found A_2_B to be significantly higher than A_2_ [5]. Usually, the frequencies of A_1_ and A_2_ phenotypes follow the Hardy-Weinberg equilibrium, but in some populations, such as blacks, Japanese, Chinese, and Indians, the frequency of the A_2_B is significantly higher than the expected frequency based on the frequency of A_2_ [12]. It could be due to the recessive nature of the *A_2_* gene compared to the *A_1_* gene, so a single *A_2_* gene and *B* gene show blood group A_2_B phenotypically, whereas two *A_2_* genes or one *A_2_* gene and one *O* gene are required for blood group A_2_ [13]. It is also postulated that the higher frequency of the A_2_B subtype in these populations could be attributed partially to the reduced synthesis of A_1_ substance by the coexisting B enzyme in heterozygous AB individuals [5]. Ogasawara et al. studied ABO alleles by using polymerase chain reaction single-strand conformation polymorphism (SSCP) and nucleotide sequence analyses. It was evident from their study that A_2_-related allele frequencies differed between A_2_ and A_2_B. A putative recombinant allele, R101, was uncommon in individuals with the A_2_ phenotype but common in those with the A_2_B phenotype. As a result of the study findings, they concluded that R101 is most probably expressed as the A_1_ phenotype in R101/O heterozygous individuals but as the A_2_ phenotype in R101/B heterozygous individuals, thus giving rise to a high frequency of A_2_B phenotypes in R101 heterozygous individuals [4]. The imbalance in the frequencies of A_2_B and A_2_ subtypes in blacks has been explained by the domination of the *B* gene on the phenol typing expression of A_1_B causing this A_1_ to be expressed as A_2_ or A_int_ leading to A_2_B excess [14,15]. We also encountered 37 cases of the A_int_ subtype, which could also be explained by the same reason.

Weaker subtypes of A usually present as group discrepancies. We encountered one case of A_end_, which is a weak subtype of blood group A. Thakral et al. found that weaker subtypes of ABO resulted in blood group discrepancies in 1:5100 donor samples in their study [16]. Shastry and Bhat, in their study of 40113 samples, found the frequency of weak A subtypes to be 0.01% [5]. All blood group discrepancies should be resolved to rule out any weaker ABO subtype. We encountered anti-A_1_ antibodies in one case in each of blood groups A_int_ and A_2_B, which were not clinically significant. If clinically significant (reacting at 37°C), they can lead to fatal transfusion reactions. Mishra et al. studied 2874 samples but found only three anti-A_1_ antibodies, none of which were clinically significant [17]. It is recommended to perform testing for anti-A_1_ antibodies in subtypes other than A_1_, especially in settings of ABO-incompatible organ transplantation.

Several case reports regarding A subtypes have been published us [18,19]; however, this is the first study related to the subtypes of blood groups A and AB in this region. The study shows that this region has a significant imbalance of A_2_ and A_2_B subtypes, which could be due to the presence of *B* gene suppressing the *A_1_* gene leading to A_int_ and A_2_B excess. Molecular studies would have helped, but they were beyond the scope of our study. With rising ABO-incompatible organ transplantations, we recommend mandatory testing of blood groups A and AB with anti-A_1_ and anti-H lectin. The importance of subtyping blood group A during incompatible organ transplantation workup has been highlighted by Sachan in a case report from South India [20]. The correct blood typing of donor and recipient samples is needed to prevent any incompatibility. The limitation of the study was that it was performed in an institutional setup, with a limited sample size. We recommend that large population-based studies should be performed in this region to understand the frequency and distribution of various subtypes of the ABO blood group.

## Conclusions

This is the first study regarding the prevalence of various subtypes of blood groups A and AB, as well as anti-A_1_ antibodies, in this region. We found a significant imbalance in A_2_ and A_2_B. We recommend immunohematological testing for subtypes and the presence of anti-A_1_ antibodies in blood groups A and AB. Population-based molecular studies are suggested to understand the prevalence of subtypes in this region.
